# Microfluidic Formation of Double-Stacked Planar Bilayer Lipid Membranes by Controlling the Water-Oil Interface

**DOI:** 10.3390/mi9050253

**Published:** 2018-05-22

**Authors:** Kan Shoji, Ryuji Kawano

**Affiliations:** Department of Biotechnology and Life Science, Tokyo University of Agriculture and Technology, 2-24-16 Naka-cho, Koganei-shi, Tokyo 184-8588, Japan

**Keywords:** microfluidic lipid bilayer formation, multiple lipid bilayers, water-oil phase flow

## Abstract

This study reports double-stacked planar bilayer lipid membranes (pBLMs) formed using a droplet contact method (DCM) for microfluidic formation with five-layered microchannels that have four micro guide pillars. pBLMs are valuable for analyzing membrane proteins and modeling cell membranes. Furthermore, multiple-pBLM systems have broadened the field of application such as electronic components, light-sensors, and batteries because of electrical characteristics of pBLMs and membrane proteins. Although multiple-stacked pBLMs have potential, the formation of multiple-pBLMs on a micrometer scale still faces challenges. In this study, we applied a DCM strategy to pBLM formation using microfluidic techniques and attempted to form double-stacked pBLMs in micro-meter scale. First, microchannels with micro pillars were designed via hydrodynamic simulations to form a five-layered flow with aqueous and lipid/oil solutions. Then, pBLMs were successfully formed by controlling the pumping pressure of the solutions and allowing contact between the two lipid monolayers. Finally, pore-forming proteins were reconstituted in the pBLMs, and ion current signals of nanopores were obtained as confirmed by electrical measurements, indicating that double-stacked pBLMs were successfully formed. The strategy for the double-stacked pBLM formation can be applied to highly integrated nanopore-based systems.

## 1. Introduction

Planar bilayer lipid membranes (pBLMs) are powerful tools for measuring ion channels [1,2], pore-forming proteins [3,4], peptides [5,6,7], and synthetic channels [8,9], owing to their transport activities that can be evaluated via electrical measurements. Furthermore, nanopore sensing with biological nanopores reconstituted into the pBLM offers an opportunity for extremely sensitive and selective biosensors by measuring the change of ion current caused by the reversible binding or translocation of a single molecule in a single nanopore. These sensors are attracting attention as a DNA sequencer [10,11] and a detection method of biomolecules [12,13] because of their high sensitivity and molecular selectively. Recently, a combination of nanopore sensing and DNA computing [14,15] and nanopore systems with multiple-stacked pBLMs [16,17,18,19] have been reported, and they led to multi-functional sensors and cascade type nanopore-based calculators. Especially, multiple-pBLM systems will broaden the field of application such as electronic components, light-sensors, and batteries because of electrical characteristics of pBLMs and membrane proteins such as high capacitance and resistance of pBLMs and permselectivity of membrane proteins [16,17,18]. We also reported a stacked biofuel cell system in which each cell is isolated by pBLMs to apply as the battery of bio-robots [17]. Although the multiple-stacked pBLMs have potential, few methods to form multiple-stacked pBLMs in micro-meter space have been considered. pBLMs are generally prepared using the Montal–Mueller method, wherein two lipid monolayers formed at the air-water interface are attached in the hole of a Teflon wall (Figure S1a). This method is very time-consuming, and the membrane is not stable. Therefore, in the last decade, numerous studies have attempted to use microfluidic technologies to improve the throughput of the pBLM formation [20,21,22,23,24,25,26]. Arrayed pBLMs are automatically formed by sequentially flowing aqueous and lipid/oil solutions through microchannels comprising a main flow channel and closed chambers (Figure S1b). Furthermore, microfluidic technologies have been applied not only to provide high-throughput platforms but also to increase the stability of the pBLMs and to reduce the noise level for electrophysiological measurements, and to be able to flush/refresh solutions [27,28,29,30,31]. However, these microfluidic methods have not been applied to the formation of multiple-stacked pBLMs. On the other hand, the droplet contact method (DCM) [22,32,33,34,35,36] has been reported to prepare stable pBLMs. Using this method, pBLMs are formed by contacting two droplets that are surrounded by a lipid monolayer (Figure S1d). The durability of the membranes formed using DCM is improved; pBLMs can be maintained for a long time (2 weeks) and are stable against mechanical vibration [36], because much of the lipid/oil solution is located around the area of lipid bilayer, and lipid molecules can be supplied and supported to the membrane region (Figure S1d). Furthermore, multiple-stacked pBLMs formed by DCMs have been reported [32,33,37,38]. Although stable pBLMs can be prepared by the DCM-based formation, DCM-based methods have some disadvantages for stacking pBLMs. First, the distance of each pBLM becomes more than hundreds of micrometers. Because the running time of nanopore-based cascade systems depends on the distance, we have to miniaturize the nanopore device to improve the running time. However, the electrodes insertion for measurements of ion currents must be required, and the size of droplets should be bigger than the one of electrodes. In fact, the size of droplets was about 500 μm in diameter, and the gap of pBLMs was also 500 μm in the DCM-based microfluidic formation of stacked pBLMs [38]. Second, the inner solution in micrometers scale droplets cannot be exchanged. Although some solution exchanges and, additionally, chemical injections would be required to develop nanopore-based systems with multiple chemical reactions and sensing, exchanging the inner solution is very difficult, because droplets are isolated from other aqueous solutions by being surrounded with an oil solution.

**Figure 1:**
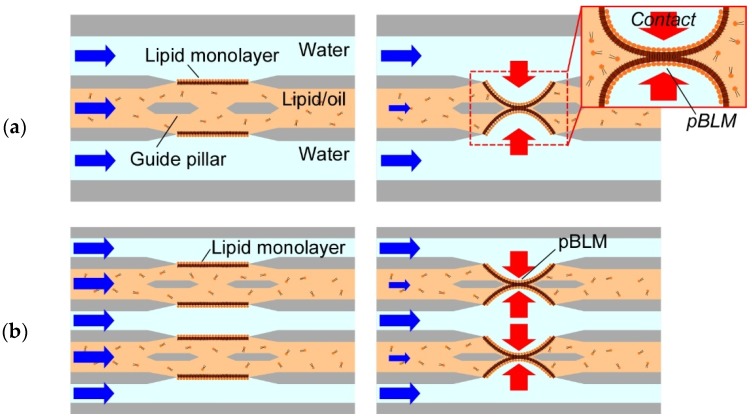
Concept of our formation method of pBLMs. (**a**) By applying the strategy of the DCM to pBLM formation using microfluidic techniques, a pBLM with open flow channels on both sides can be prepared. (**b**) Double-stacked pBLMs can be formed using our method.

Therefore, we propose an approach for pBLM formation based on the strategy of lipid monolayer contacting in a microfluidics system (Figure 1a) and present a method for the double-stacked pBLM formation in a microfluidic device by optimizing the geometry of the guide pillars (Figure 1b). This method promises to prepare narrower gapped pBLM stacks than conventional droplet-based methods and pBLMs with open flow channels on both sides. To construct double-stacked pBLMs, first, we proposed to build guide pillars for the formation of a five-layered flow comprising aqueous and lipid/oil solutions. Next, we designed the microchannel with guide pillars using a hydrodynamic simulation. Then, we formed double-stacked pBLMs in a five-layered microchannel by decreasing the flow pressure after the five-layered flow was established. Formation of stable pBLMs in microchannels allowed us to form double-stacked pBLMs. Finally, we reconstituted pore-forming proteins in the pBLMs and measured the channel current through the two membranes.

## 2. Materials and Methods

### 2.1. Regents and Chemicals

1,2-Diphytanoyl-sn-glycero-3-phosphocholine (DPhPC; Avanti Polar Lipids, Alabaster, AL, USA) and hexadecane (Wako Pure Chemical Industries, Ltd., Osaka, Japan) were used as the lipid/oil solution. Potassium chloride (KCl; Nacalai Tesque, Kyoto, Japan) and 3-morpholinopropane-1-sulfonic acid (MOPS; Nacalai Tesque, Kyoto, Japan) were used as the aqueous solution (1.0 M KCl, 10 mM MOPS, pH 7.0). The MOPS solution was prepared using ultrapure water from a Milli-Q (Merck Millipore Corp., Billerica, MA, USA). Alpha-hemolysin (αHL; Sigma-Aldrich, St. Louis, MO, USA) was obtained as a monomer protein isolated from Staphylococcus aureus in the form of a powder, dissolved at a concentration of 1 mg/mL in ultrapure water, and stored at −80 °C. For use, samples were diluted in the designated concentration using a buffered electrolyte solution and stored at 4 °C. SU-8 3025 (MicroChem Corp., Newton, MA, USA) was used as a photoresist. Polydimethylsiloxane (PDMS; Sylgard 184, Dow Corning Toray Co., Ltd., Tokyo, Japan) was used for the material of microchannels.

### 2.2. Concept of Double-Stacked pBLM Formation Using Five-Layered Microchannels

**Figure 2:**
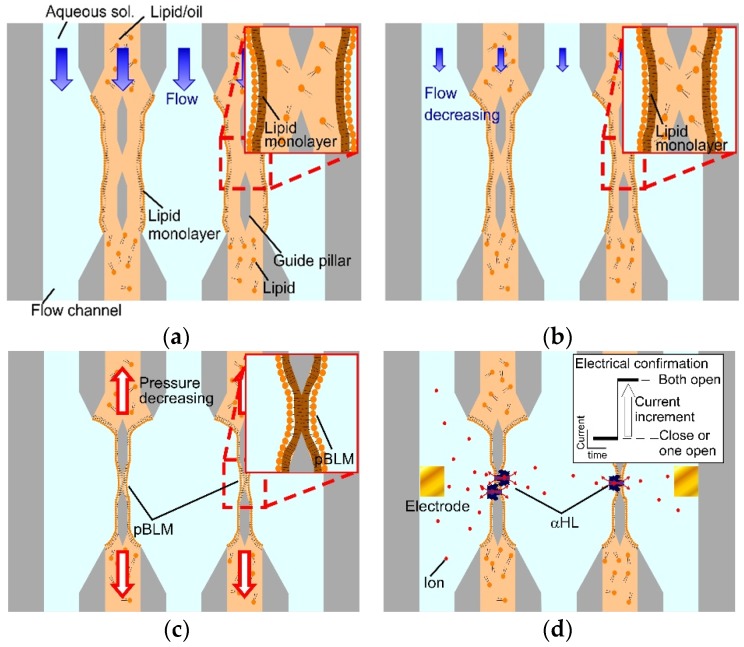
Schemes of double-stacked pBLM formation processes using the five-layered microchannel with guide pillars. (**a**) Aqueous solutions and lipid/oil solutions flow alternately in each channel, and the five-layered flow is formed. (**b**) The flow pressure of all the solutions is decreased. (**c**) Lipid monolayers are contacted by decreasing the flow pressure of lipid/oil solutions, and pBLMs are formed. (**d**) Pore-forming proteins are reconstituted to the pBLMs, and the channel current through the paralleled pBLMs can be measured.

The five-layered microchannels comprised five inlets, five outlets, area confluent area joining all channels, and guide pillars in the confluent area. In the microchannels, double-stacked pBLMs are formed as follows. First, the aqueous and lipid/oil solutions flow to each channel to form a five-layered flow with three aqueous solution layers and two lipid/oil solution layers in the confluent area (Figure 2a). At that time, lipid monolayers spontaneously form at the interfaces between the aqueous and lipid/oil solutions. Then, the flow pressures decrease to stabilize the interfaces between the aqueous and lipid/oil solutions (Figure 2b). Finally, the lipid monolayers are contacted in the gaps of pillars by reducing the flow pressure of the lipid/oil solutions (Figure 2c). As a result, pBLMs are formed, and membrane proteins can be reconstituted to the pBLMs (Figure 2d). To form double-stacked pBLMs using the above method, we set micro guide pillars with hydrophobicity at the midpoints of the channel for increasing the area of the hydrophobic surface. The guide pillars have a dual function as follows. One is to guide the lipid/oil solution along their surface because of their hydrophobicity. Second is the aperture for the contact area of two lipid monolayers. Restricting the contact area of the two lipid monolayers may increase the stability of the BLMs.

### 2.3. Hydrodynamic Simulation of Five-Layered Microchannels

The behaviors of aqueous and lipid/oil solutions in the confluent area with and without the guide pillars were simulated with COMSOL Multiphysics 4.2a (COMSOL Inc., Burlington, MA, USA) using the microfluidics module to ascertain the effects of the guide pillars for multi-layered flow formation. The density and viscosity of the aqueous and lipid/oil solutions were set to 1.0 g/mL and 1.0 mPa·s and 0.77 g/mL and 3.34 mPa·s, respectively. The boundary tension between the aqueous and lipid/oil solutions was 15 mN/m [39]. The contact angle between the channel and the aqueous solution was 3/4 π. The contact angle was determined by observing the interface between aqueous and lipid/oil solutions in a PDMS microchannel. The flow rate was estimated with the condition of 20 mbar flow pressure and set to 10 μL/h for all channels. The flow behaviors of the aqueous and lipid/oil solutions were simulated with and without guide pillars, and the results were compared. Furthermore, the dependency of flow behaviors on the guide pillar size was simulated for designing the guide pillars.

### 2.4. Fabrication of Five-Layered Microchannels

**Figure 3:**
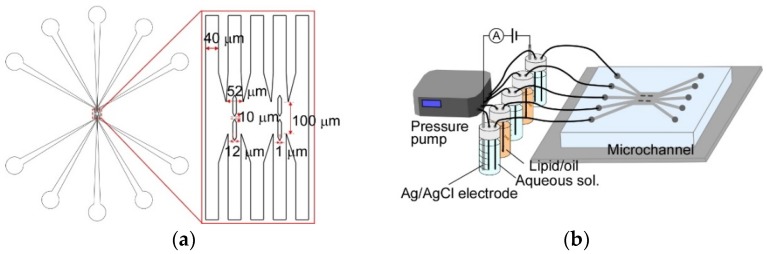
(**a**) Design of the five-layered microchannel. (**b**) Experimental setup for double-stacked pBLM formation.

The microchannels were fabricated by standard photolithography techniques. SU-8 3025 was used as the master photoresist. Film masks were fabricated with a spot diameter and drawing pitch for a laser of 4 and 1 μm, respectively, for the photomasks. An exposure masking system (UV-KUB 2, KLOE, Montpellier, France) was used for UV exposing. The channels were fabricated by PDMS molding. PDMS was cured for 2 h at 95 °C. The PDMS channels were bonded to a cover glass (NEO cover glass, Matsunami Glass Ind., Ltd., Osaka, Japan) using plasma etching equipment (FA-1, Samco Inc., Kyoto, Japan). The designed values are shown in Figure 3 and Figure S3. The width of each flow channel was 40 μm. The width and length of the confluent area were 360 and 100 μm, respectively. The tips of guide pillars were designed 1 mm flat shape. The height of all channels was 20 μm.

### 2.5. Microfluidic Experiment of Double-Stacked pBLM Formation

**Figure 4:**
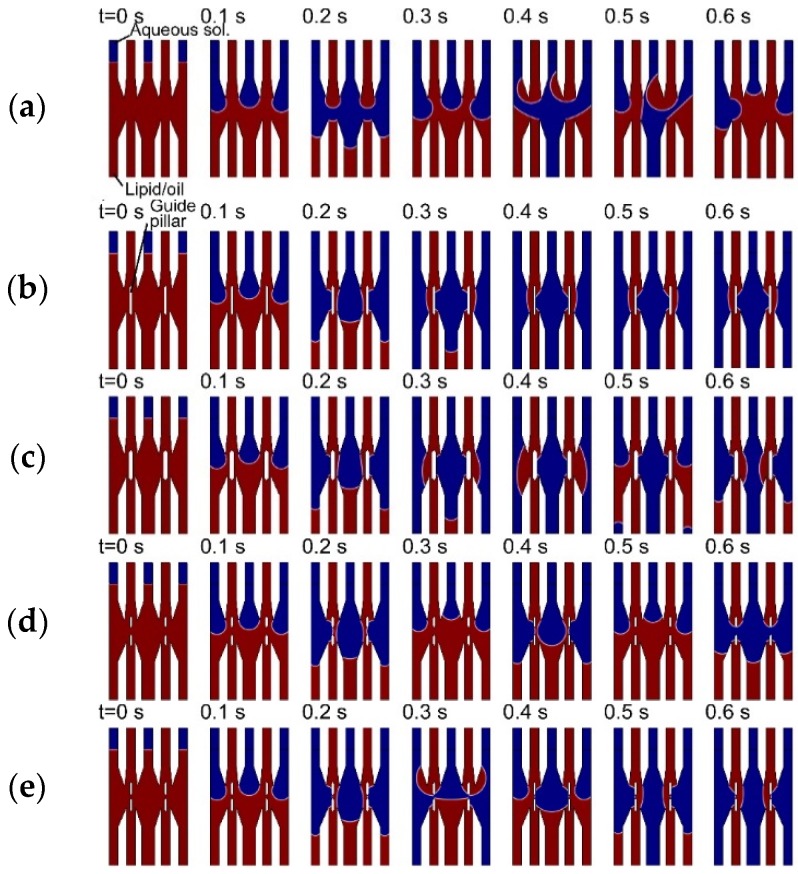
Simulation results of microfluidic behaviors (**a**) without guide pillars and with guide pillars with a width of (**b**) 10 μm, (**c**) 20 μm, (**d**) 10 μm and a gap of 30 μm, and (**e**) 10 μm and with a gap of 10 μm). In the conditions of (**a**,**c**,**d**), all solutions were jumbled. In the conditions of (**b**,**e**), all solutions flowed straight, and a five-layered flow was formed. Blue and red colors show the aqueous and lipid/oil solutions.

The experimental setup for the double-stacked pBLM formation and the photograph and the microscopic image of the five-layered microchannel are shown in Figure 3b and Figure 4a. DPhPC/hexadecane (1 mM) was prepared as the lipid/oil solution, and 1 M KCl (10 mM MOPS, pH 7.0) was used as the aqueous solution. Each solution flowed with a pressure pump (MFCS-EZ, Fluigent, Paris, France). The PDMS microchannels were coated with a fluorochemical coating solution (NovecTM 1720, 3 M, St. Paul, MN, USA) to uniformize the surface properties of the PDMS and glass surfaces in the microchannel and prevent the adherence of lipid molecules to the surface of the microchannel. The microchannels were filled with the solution by insertion with a micropipette and dried at 100 °C for 20 min. The contact angles of the aqueous solution and the PDMS surface before and after coating were 107° and 105°, respectively. The one of the DPhPC/hexadecane and the PDMS surface before and after coating were 26° and 27°, respectively.

First, each solution was flowed to form a five-layered flow at 250 mbar of pumping pressures. After confirming the formation of five-layered flow, all pumping pressures were gradually reduced to 20 mbar while maintaining the five-layered flow. Finally, the pumping pressure of the aqueous solutions was fixed at 20 mbar, and the pumping pressure of the lipid/oil solutions was gradually reduced until interfaces were contacted on the gap of pillars. All pressure control was carried out manually. Microchannel observation was performed using an inverted microscope (AXJ-5300TPHFL, WRAYMER Inc., Osaka, Japan) mounted with a microscopic digital camera (FLOYD, WRAYMER Inc., Osaka, Japan).

### 2.6. Reconstitution of Membrane Protein to the Double-Stacked pBLMs

To confirm the formation of double-stacked pBLMs, pore-forming membrane proteins were reconstituted in the double-stacked pBLMs. αHL was used as the pore-forming membrane protein. Moreover, 1 M KCl (10 mM MOPS, pH 7.0) containing 10 nM αHL and 1 mg/mL bovine serum albumin (BSA; Sigma-Aldrich, St. Louis, MO, USA) was used for all aqueous solutions. BSA was utilized for preventing the adhesion of proteins in the microchannel. DPhPC/hexadecane (1 mM) was used as the lipid/oil solution. Ag/AgCl electrodes were used for channel current recording, and the electrodes were inserted into the reservoir of the pressure pump. Channel currents at +120 mV were monitored using a patch-clamp amplifier (Pico 2, Tecella, Foothill Ranch, CA, USA) with a 7.9 kHz low-pass filter at a sampling frequency of 40 kHz.

## 3. Results and Discussion

### 3.1. Hydrodynamic Simulation for Designing the Double-Stacked BLMs

To confirm the effect of guide pillars for formation of the five-layered flow and design the geometries of the microchannels, a hydrodynamic simulation for the microfluidic behaviors of the aqueous and lipid/oil solutions was performed, because the stable five-layered flow of the aqueous and lipid/oil solutions is very important as the pre-state for the stacked pBLM formation in our method. First, we confirmed the microfluidic behaviors with and without guide pillars, as shown in Figure 4 and Video S1. Without guide pillars, all solutions did not flow straight and flowed to the other channels, because the surface tension between the aqueous (blue) and lipid/oil (red) solutions is different (Figure 4a). Under microfluidic experiments, the surface tensions strongly influence the fluidic behaviors compared to macro-scale conditions. To configure a straight-flowing lipid/oil solution, we set the guide pillars at the midpoints of the channels for increasing the area of hydrophobic surfaces (Figure 4b). With guide pillars, the lipid/oil solutions flowed along the guide pillars, and a five-layered flow was formed (Figure 4b). Because of the hydrophobicity of the guide pillars, the surface force between the guide pillars and the lipid/oil solutions becomes strong, resulting in the straight-flowing path to be maintained. Some groups reported the two or three-layered flowing with aqueous and oil solutions by modifying the surface [40] or by fabricating grooves in the channels [41]. By comparing with these reports, we can fabricate our devices much more simply.

Next, we simulated the geometry of the guide pillars, including the width and gap distance. The pillar width was changed from 10 to 15 μm and from 15 to 20 μm (Figure 4c). In the case of 20 μm width, the straight-flowing lipid/oil solution was disturbed (0.4 to 0.5 s in Figure 4c), because the width was very large. In a similar fashion, straight flow was not observed in the case of a 15 μm width pillar. From these results, the pillar width should be designed to 10 μm. The gap distance is also important, because this is where the pBLM is formed; we changed the gap distance from 5 to 10 μm and from 10 to 30 μm. Figure 4d,e shows the results of the 30 and 10 μm gap system. With a 30 μm pillar gap, aqueous solution layers break through the lipid/oil layer at the gap; therefore, five-layered flows were not maintained (Figure 4d). With the 10 μm pillar gap, although the aqueous layers broke the lipid/oil layer at the gap in the initial stage, the five-layered flow formed and was maintained with continuing flow (Figure 4e). As expected, with a 5 μm pillar gap, a five-layered flow also formed. From these results, we decided on the use of pillar width and gap distances of 10 μm.

### 3.2. Formation of Double-Stacked pBLMs Using Five-Layered Microchannels

**Figure 5:**
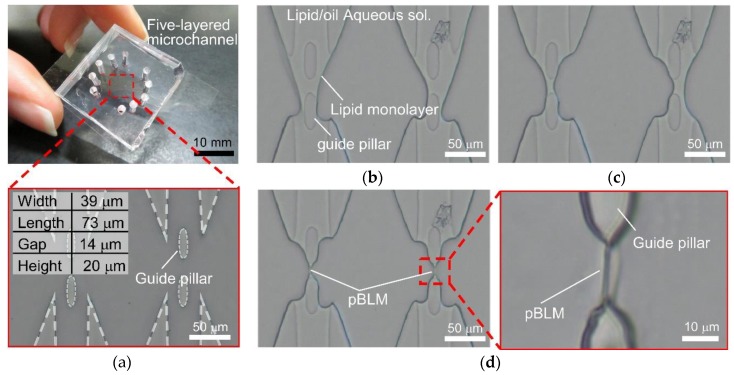
Photograph and microscopic images of the microchannel. (**a**) The photograph of the five-layered microchannel. The insert shows the microscopic image of the confluent area of the channel. The five-layered flow of aqueous and lipid/oil solutions at (**b**) high pumping pressure (>200 mbar) and (**c**) low pumping pressure (<20 mbar). (**d**) The parallel array of planar lipid bilayers formed in the micro space. The inset shows the enlarged photograph of the lipid bilayer.

Based on the optimal conditions determined from the hydrodynamic simulation, we attempted to form double-stacked pBLMs using five-layered microchannels (Figure 5 and Video S2). First, a five-layered flow comprising three aqueous and two lipid/oil solution layers was formed using the microfluidic device (Figure 5a,b). After the five-layered flow formed, the flow speed of all channels was reduced by decreasing the pumping pressure from 200 to 20 mbar for stabilizing the interfaces (Figure 5c). At that time, the width of the lipid/oil solution decreased in the gap area, and the interfaces seemed to be stable. Then, the two aqueous–oil interfaces in the gap were contacted and formed a lipid bilayer (as confirmed in the next section) by decreasing the pumping pressure of lipid/oil solutions (Figure 5d). Furthermore, when we changed the number of pillars from 2 to 4, five-layered flow and double-stacked pBLMs were formed (Figure S4, Video S3). While it is very difficult to observe the surface of pBLMs in our system, because the pBLMs were vertically placed, we expected that the shape of pBLMs is almost square and the pBLMs are surrounded by annulus by reference to a previous work to form the free-standing pBLM by using a microchannel [31]. By contrast, without guide pillars, five-layered flow was not formed as expected from the simulation. Previously, some double-stacked pBLMs have been reported using the droplet-based method in microchannels [32,33,37,38]; our method has significant advantages (the shorter gap distance (150 μm) of each pBLM is obtained, and open flow channels are constructed on both sides of pBLMs). First, we discussed the success rate of the stacked pBLM formation. The success rate to form double-stacked pBLMs was around 50%. Although the five-layered flow can be formed with 100% success rate, almost all failures were raised when interfaces were contacted. In this study, we could not discuss the shape of the pillar edge because of the lower spatial resolution of the manufacturing systems. To improve the success rate, the shape of pillar should be optimized. However, we can easily and rapidly reattempt to form double-stacked pBLMs by flashing all solutions at high pumping pressure flowing.

Next, the lifetime of the pBLM was examined. The pBLM remained at the interspace between guide pillars for ca. 2 h (Figure S5). Compared with other methods for the pBLM formation such as the millimeter scale DCM (2 weeks) [36], the droplet-based method in microfluidic channels (12 h) [38] and freestanding pBLMs (36 h) [30], the lifetime of the pBLMs was shorter. Because the solutions at both sides of pBLMs continuously flow and the shape of the pillar tips could not be controlled due to the low spatial resolution of the photolithography instruments, the shorter lifetime was obtained. However, the stacked pBLMs that are placed in a micro space and have flow channels on their both sides have never been reported. To improve the lifetime of stacked pBLMs, improvement of fluidic controllability at low flow speed area and higher spatial resolution to get the designed shape of pillars are required using state-of-the-art equipment.

### 3.3. Channel Current Measurement of Nanopores Reconstituted in the Double-Stacked BLMs

**Figure 6:**
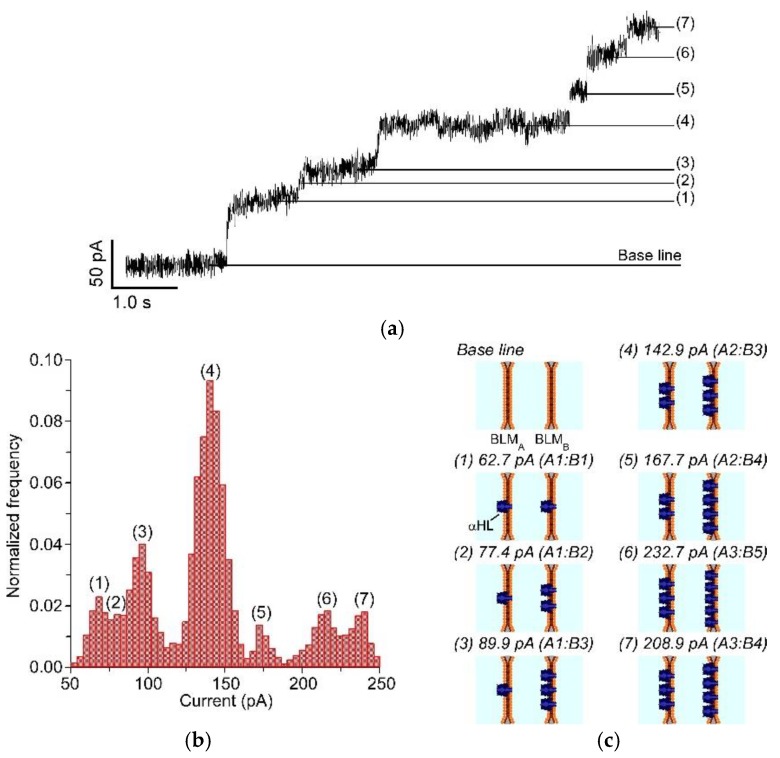
(**a**) Ion current signals of αHLs reconstituted in the double-stacked pBLMs. The channel current signal was 0 pA before the double-stacked pBLM was formed. Then, the ion current increased by reconstituting αHLs in the double-stacked pBLMs, and seven step signals were obtained. (**b**) Histogram of the ion current. (**c**) Schematic of the estimated pore conditions in each step.

**Figure 7:**
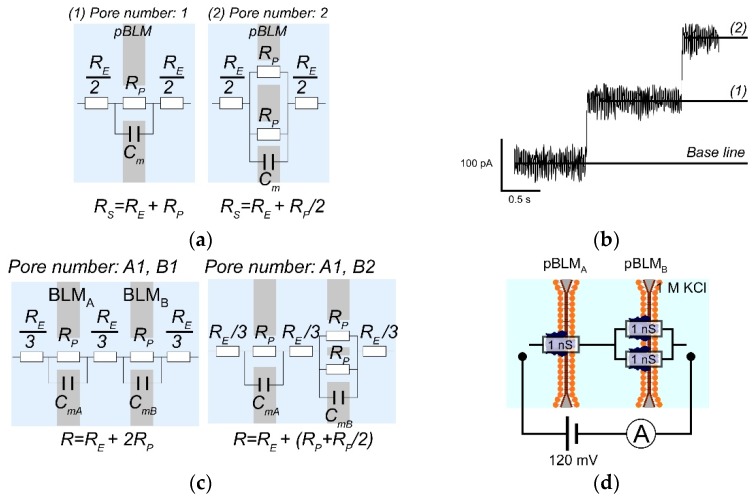
(**a**) Schematic and electrical circuits of the single nanopore system. (**b**) Ion current signals of αHLs reconstituted to the single pBLM. The current steps were almost 120 pA. (**c**) Schematics and electrical circuits of the double-stacked pBLMs with nanopores. (**d**) A schematic and electrical circuit of the double-stacked pBLMs with αHLs under the condition of 1 M KCl solution and 120 mV application.

We demonstrated channel current measurements of αHL through double-stacked pBLMs (Figure 6 and Figure S6) to confirm the formation of pBLMs. Although the formation of pBLMs is usually confirmed by measuring membrane capacitances of pBLMs, we evaluated the formation of pBLMs by the reconstitution of αHL pores, because it is difficult to show the doubled-stacked structure from the membrane capacitance. Initially, the current was 0 pA when both or one of the pBLMs were not formed. Then, when both pBLMs formed and αHLs were reconstituted in the double-stacked pBLMs, step-like currents were clearly observed, as shown in Figure 6a. One step indicates a single αHL reconstitution. In our systems, current can be observed when αHLs form pores in both pBLMs [38]. Next, we estimated the number of pores in each pBLM using the model of equivalent circuit (Figure 7). In the case of a single pBLM with αHL pores, the electrical circuit of the nanopore system is shown in Figure 7a, and the total resistance (R_S_) of the single pBLM with nanopores is calculated using Equation (1).

(1) $$R_{S}=R_{E}+R_{P}/n$$

In Equation (1), R_E_ and R_P_ indicate the resistance of the electrolysis solution and a single nanopore, respectively, and n is the number of pores. Because the resistance of electrolysis solution is sufficiently smaller than that of one nanopore, R_E can be ignored. Commonly, the αHL pore has a constant conductance of 1 nS in 1 M KCl, resulting in each step of the ion currents being almost constant at 120 pA under application of 120 mV. As a result of checking the conditions in this study, the ion current steps were also shown to be almost 120 pA from a single pBLM formed by a three-layered microchannel (Figure 7b and Figure S7). On the other hand, in the case of double-stacked pBLMs with αHL pores, the step values were not constant. An equivalent circuit can predict this phenomenon, described as follows (Figure 7c). The total resistance in the double-stacked pBLMs with nanopores is shown in Equation (2).

(2) $$R=R_{E}+\left(\frac{R_{P}}{n_{A}}+\frac{R_{P}}{n_{B}}\right)$$

In Equation (2), n_A_ and n_B_ are the number of pores in pBLMa and pBLMb, respectively; R_E_ can be ignored as in Equation (1), and αHL shows 1 nS conductance (Figure 7d). Using Equation (2), the ion current that depends on the number of pores can be calculated, and the inconsistent step current can be explained by how many αHL reconstituted in two different pBLMs (see detailed description in Table S1). From these calculation results, we estimated the pore number in each pBLM as shown in Figure 6b,c. In the estimation of pore formation, single pores were initially reconstituted in both pBLMs, and ~60 pA current was obtained. Then, pores were randomly reconstituted one-by-one in each of the pBLMs, as shown in Figure 6c. The estimated currents from the model circuit could be predicted well using the experimental results.

## 4. Conclusions

In conclusion, we proposed a new approach for a pBLM formation in which the DCM strategy is applied to the microfluidic formation of pBLMs. We achieved double-stacked pBLMs with open flow channels on both sides by using a five-layered microchannel comprising aqueous and lipid/oil solutions. First, we designed five-layered microchannels with hydrophobic guide pillars in the middle area for formation of five-layered flow using hydrodynamic simulations. The formation of five-layered flow was confirmed by microfluidic experiments. Then, double-stacked pBLMs were successfully formed by contacting the lipid monolayers in the interfaces between the aqueous and lipid/oil layers. We also successfully reconstituted membrane proteins in the pBLMs and measured the ion currents through the two membranes with nanopores. The ion currents can be estimated from the electrical circuit of the nanopore system, and the measured ion currents result in the same values as the calculated currents. We think that our double-stacked pBLM system has the potential to develop high speed and multiple functional nanopore calculators with multiple pBLMs.

## Supplementary Materials

The following are available online at http://www.mdpi.com/2072-666X/9/5/253/s1, Figure S1: Methods for the formation of pBLMs. (a) The Montal–Mueller method, wherein pBLMs are formed by attaching the lipid monolayers in the hole of a Teflon wall. (b) Conventional pBLM formation method using microfluidic techniques, wherein aqueous and lipid/oil solutions flow sequentially through the microchannel with small chambers. (c) The pBLMs cannot be paralleled because of the pressure difference between each flow channel. (d) DCM, in which the pBLM is formed by contacting two droplets surrounded with a lipid monolayer. The pBLM is supported by the lipid/oil solution, and lipid molecules are supplied to the pBLM; Figure S2: microscopic images of the microfluidic experiment for the formation of double-stacked pBLMs using the conventional microfluidic method. These solutions did not flow straight because of the channel resistance and boundary tension between aqueous and lipid/oil solutions. Scale bars are 50 μm; Figure S3: designs of five-layered microchannels with eight guide pillars; Figure S4: microscopic images of (a) the microchannel with eight guide pillars and (b) the vertical paralleled pBLMs formed by the channel; Figure S5: microscopic images of a pBLM (a) before and (b) after 2 h from the pBLM formation; Figure S6: ion current signals of αHL reconstituted in the vertical paralleled pBLMs. The ion current was changed depending on the pore number in each pBLM; Figure S7: microscopic images of (a) the three-layered microchannel and (b) pBLMs formed by the channel; Table S1: Estimated ion current of the nanopores in a double-stacked pBLM. The conductance of nanopores is 1 nS and the applied voltage is 120 mV (Figure 7d); Video S1: hydrodynamic simulations of microfluidic behaviors with and without guide pillars; Video S2: formation of the double-stacked pBLMs using the five-layered microchannel with four guide pillars; Video S3: formation of the double-stacked pBLMs using the five-layered microchannel with eight guide pillars.
