# Supplementary material for: Microfluidic Formation of Double-Stacked Planar Bilayer Lipid Membranes by Controlling the Water-Oil Interface
Source: Micromachines (Basel). 2018 May 22;9(5):253. doi: 10.3390/mi9050253 (PMC6187563; doi:10.3390/mi9050253)
Supplement: Supplementary file 1 [file micromachines-09-00253-s001.zip › micromachines-300580-supplementary/micromachines-300580-Supplementary.docx]

**Supplementary Materials: Microfluidic Formation of Double-stacked Planar Bilayer Lipid Membranes by Controlling the Water–Oil Interface**


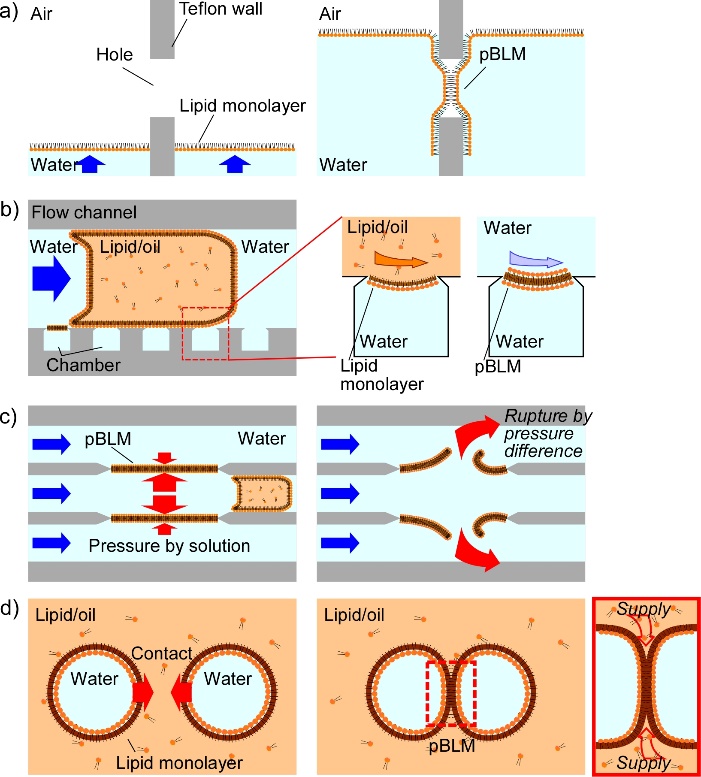


**Figure S1.** Methods for the formation of pBLMs. (**a**) The Montal–Mueller method, wherein pBLMs are formed by attaching the lipid monolayers in the hole of a Teflon wall. (**b**) Conventional pBLM formation method using microfluidic techniques, wherein aqueous and lipid/oil solutions flow sequentially through the microchannel with small chambers. (**c**) The pBLMs cannot be paralleled because of the pressure difference between each flow channel. (**d**) DCM, where the pBLM is formed by contacting two droplets surrounded with a lipid monolayer. The pBLM is supported by the lipid/oil solution, and lipid molecules are supplied to the pBLM.


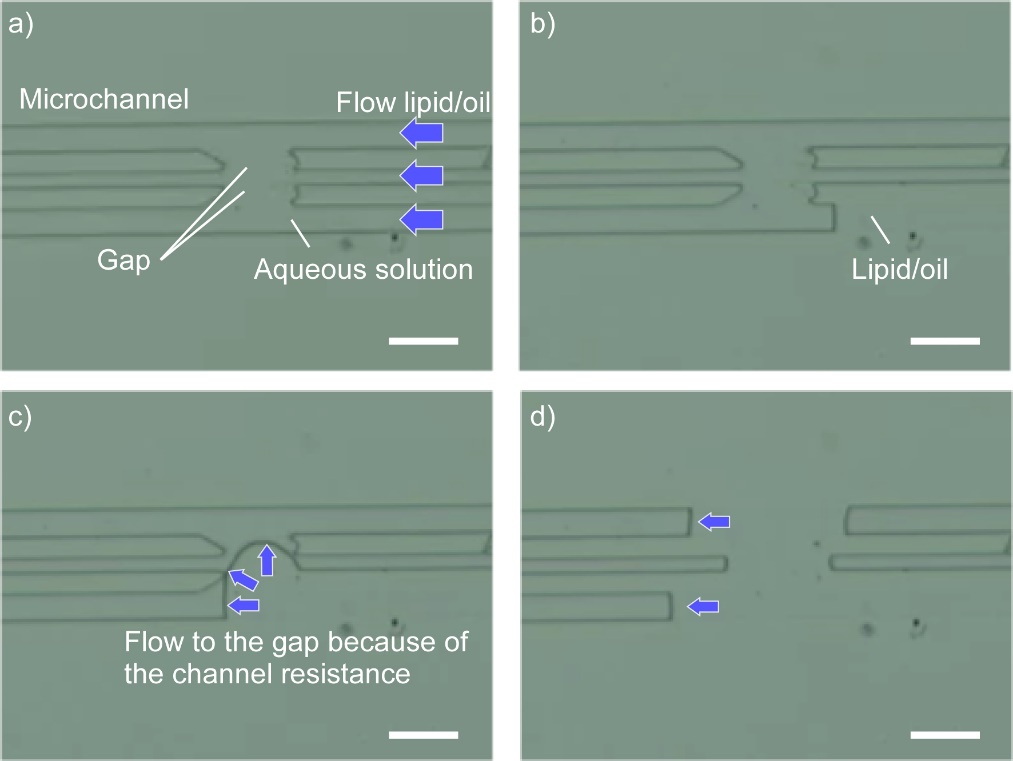


**Figure S2.** Microscopic images of the microfluidic experiment for the formation of double-stacked pBLMs by the conventional microfluidic method. These solutions did not flow straight because of the channel resistance and boundary tension between aqueous and lipid/oil solutions. Scale bars are 50 μm.


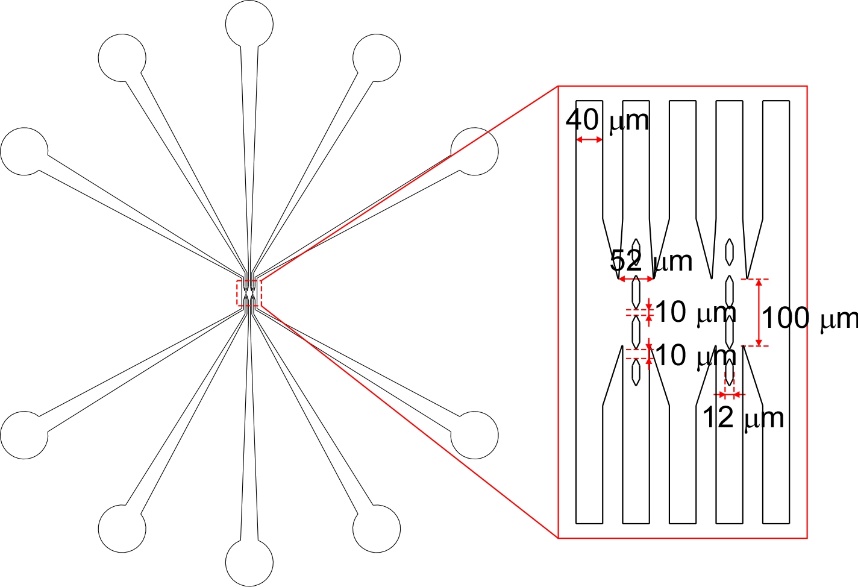


**Figure S3.** Designs of five-layered microcahnnels with eight guide pillars.


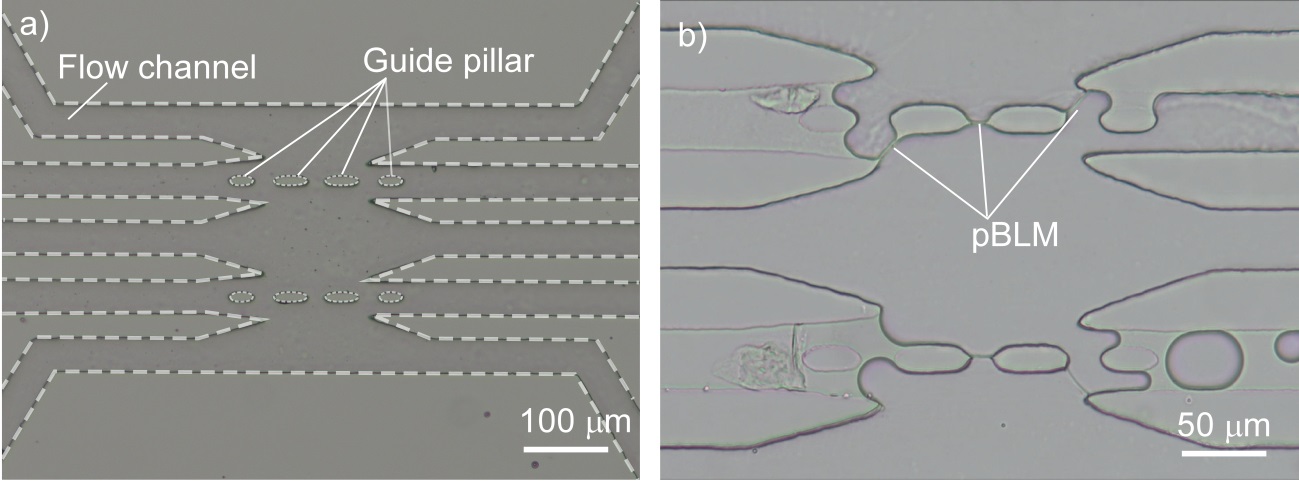


**Figure S4.** Microscopic images of (**a**) the microchannel with eight guide pillars and (**b**) the double-stacked pBLMs formed by the channel.


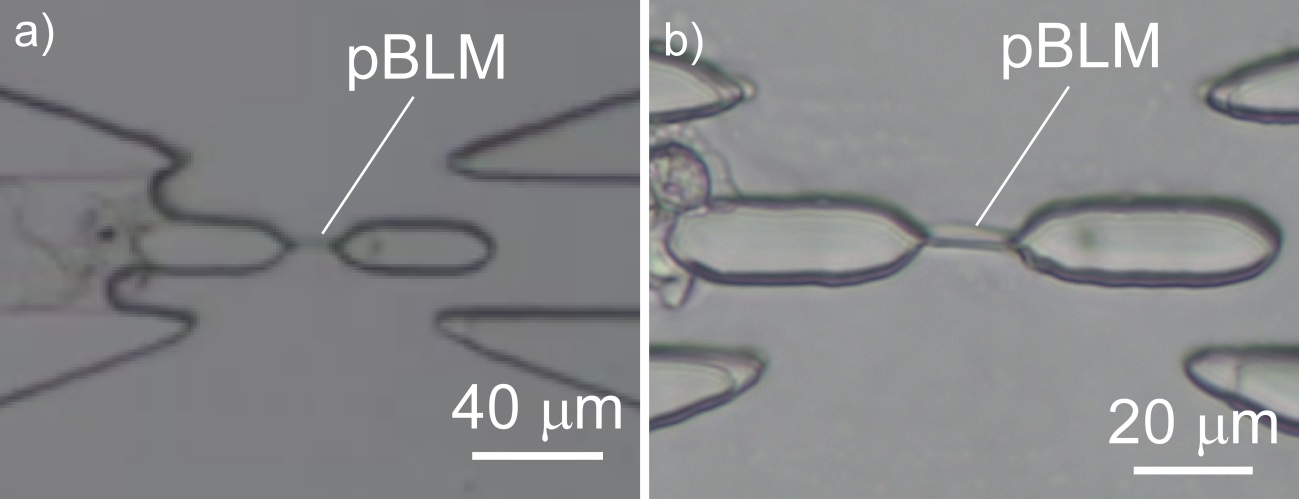


**Figure S5.** Microscopic images of a pBLM (**a**) before and (**b**) after 2 hours from the pBLM formation.


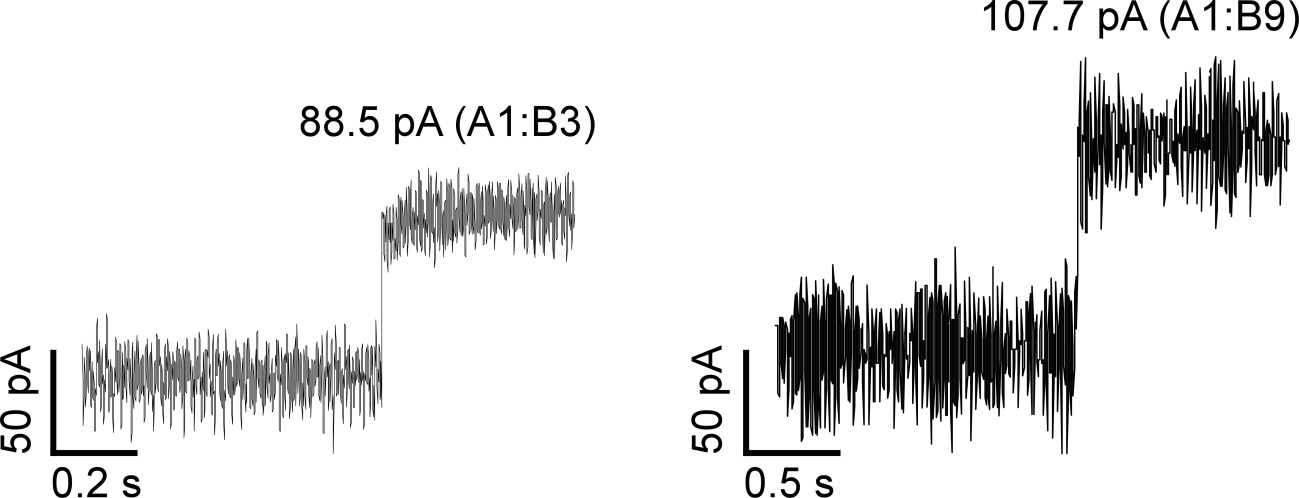


**Figure S6.** Ion current signals of αHL reconstituted in the double-stacked pBLMs. The ion current was changed depending on the pore number in each pBLM.


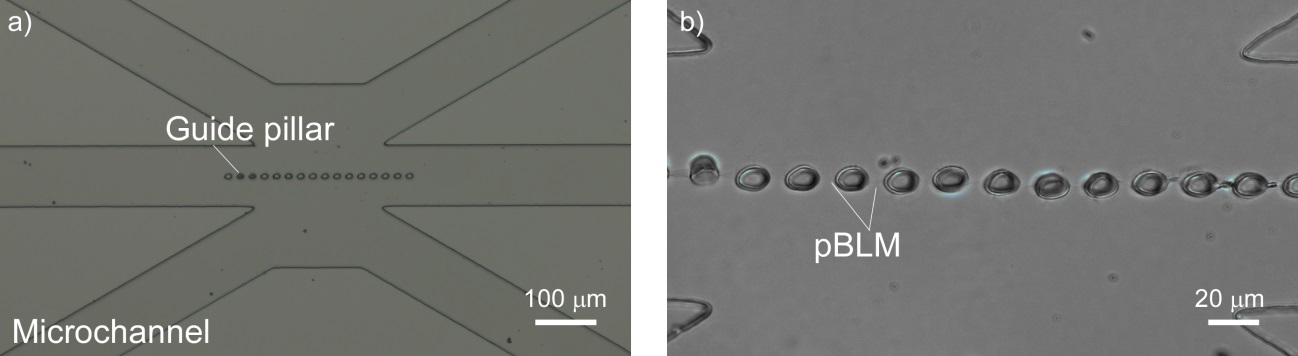


**Figure S7.** Microscopic images of (**a**) the three-layered microchannel and (**b**) pBLMs formed by the channel.

**Table S1.** Estimated ion current of the nanopores in a double-stacked pBLM. The conductance of nanopores is 1 nS and the applied voltage is 120 mV (Figure 7d).

| **Pore Number: A** | **Pore Number: B** | **Current (pA)** | **Total Pore Number** | **Pore Number: A** | **Pore Number: B** | **Current (pA)** | **Total Pore Number** |
| --- | --- | --- | --- | --- | --- | --- | --- |
| 1 | 1 | 60 | 2 | 2 | 1 | 80 | 3 |
| 1 | 2 | 80 | 3 | 2 | 2 | 120 | 4 |
| 1 | 3 | 90 | 4 | 2 | 3 | 144 | 5 |
| 1 | 4 | 96 | 5 | 2 | 4 | 160 | 6 |
| 1 | 5 | 100 | 6 | 2 | 5 | 171.4 | 7 |
| 1 | 6 | 105 | 7 | 2 | 6 | 180 | 8 |
| 1 | 7 | 106.7 | 8 | 2 | 7 | 186.7 | 9 |
| 1 | 8 | 108 | 9 | 2 | 8 | 192 | 10 |
| 1 | 9 | 109 | 10 | 2 | 9 | 196.4 | 11 |
| **Pore Number: A** | **Pore Number: B** | **Current (pA)** | **Total Pore Number** | **Pore Number: A** | **Pore Number: B** | **Current (pA)** | **Total Pore Number** |
| 3 | 1 | 90 | 4 | 4 | 1 | 96 | 3 |
| 3 | 2 | 144 | 5 | 4 | 2 | 160 | 4 |
| 3 | 3 | 180 | 6 | 4 | 3 | 205.7 | 5 |
| 3 | 4 | 205.7 | 7 | 4 | 4 | 240 | 6 |
| 3 | 5 | 225 | 8 | 4 | 5 | 266.7 | 7 |
| 3 | 6 | 240 | 9 | 4 | 6 | 288 | 8 |
| 3 | 7 | 252 | 10 | 4 | 7 | 305.5 | 9 |
| 3 | 8 | 261.8 | 11 | 4 | 8 | 320 | 10 |
| 3 | 9 | 270 | 12 | 4 | 9 | 332.3 | 11 |

Video S1: Hydrodynamic simulations of microfluidic behaviors with and without guide pillars.

Video S2: Formation of the double-stacked pBLMs by using the five-layered microchannel with four guide pillars.

Video S3: Formation of the double-stacked pBLMs by using the five-layered microchannel with eight guide pillars.
